# Supplementary material for: BBIBP-CorV Vaccination against the SARS-CoV-2 Virus Affects the Gut Microbiome
Source: Vaccines (Basel). 2023 May 4;11(5):942. doi: 10.3390/vaccines11050942 (PMC10223200; doi:10.3390/vaccines11050942)
Supplement: Supplementary file 1 [file vaccines-11-00942-s001.zip › vaccines-2359210-supplementary.pdf]

Supplementary materials

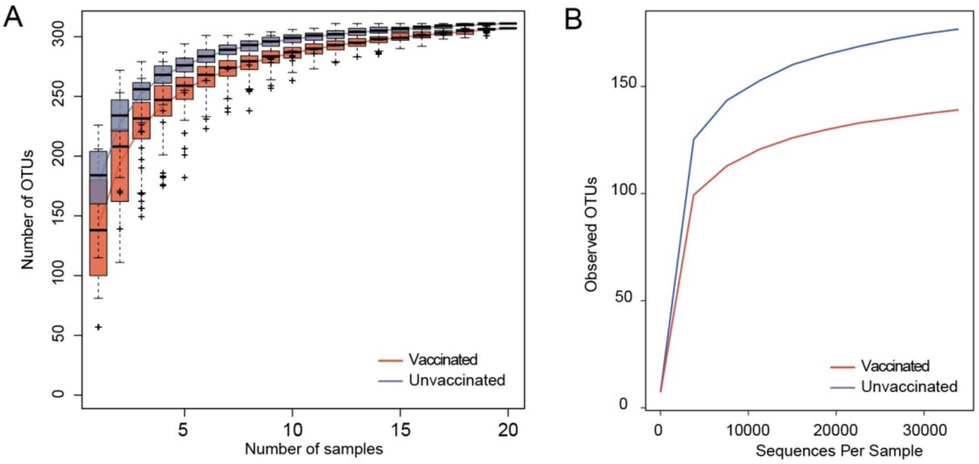

Figure S1. Rarefaction curves validating the adequate sample size and sequencing depth in each group. (A) Rarefaction curves were constructed by randomly sampling various individuals from the cohort and examining numbers detected in these individuals. When the curves were flat, they indicated that the sample size was sufficient and reasonable. **(B)** Rarefaction curves performed when gradually expanding the sequencing depth by random sampling. When the curves approach saturation as the sample sequencing depth increases, the amount of sequencing data is sufficient and stable.

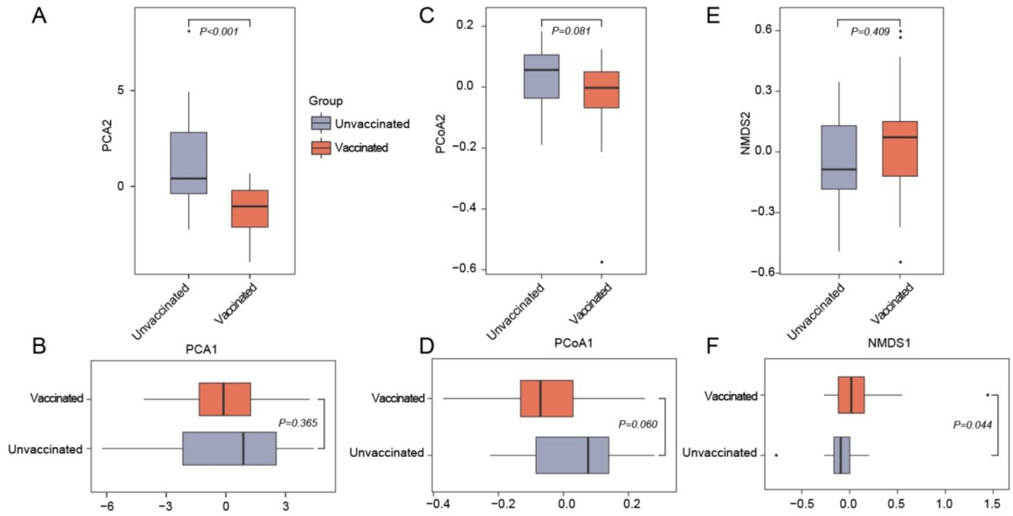

Figure S2. The distribution of β-diversity plots at axes based on genera profiles in vaccinated and unvaccinated participants. (A-B) β-diversity scatter plots distribution at the first and second PCA axes. P=0.365 for PCA1 axis; P<0.001 for PCA2 axis. (C-D) Elevation for the distribution of samples at the first and second PCoA axes. P=0.06 for PCoA1 axis; P=0.081 for PCoA2 axis. (E-F) Comparison of the features of each group at the first and second NMDS axes. P=0.044 for the NMDS1 axis; P=0.041 for the NMDS2 axis. The boxes represent the interquartile ranges, the inside lines represent the median, and the points are outliers. P-values were obtained using pairwise Wilcoxon tests.

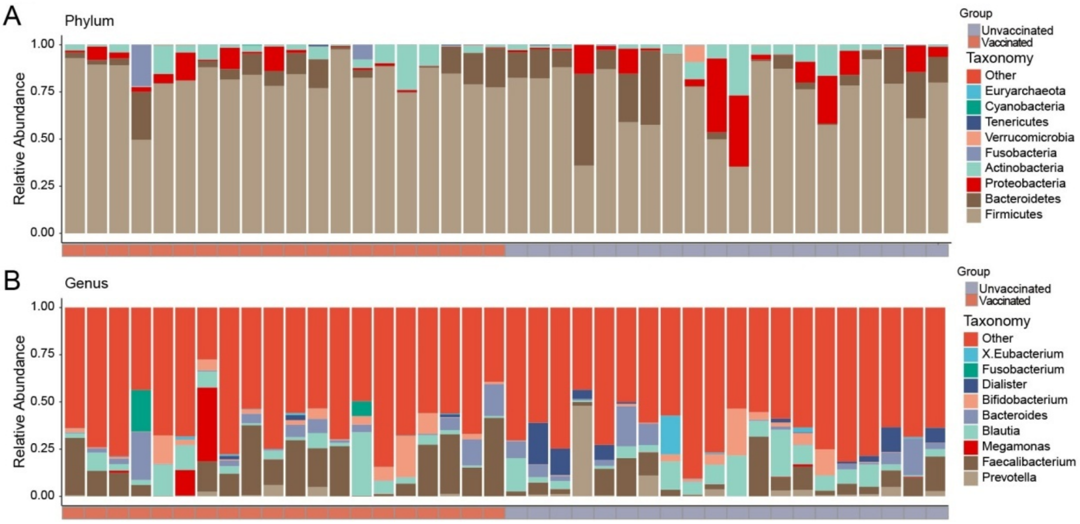

Figure S3. Relative abundance of the top 10 phyla and genera annotated in the stool of each sample. (A) Bar plots showing the relative abundance and proportion of the top 10 most abundant phyla in each sample. **(B)** The relative abundance and proportion of the top 10 most dominant genera are described in each sample. Other in Figure S3A and S3B indicate the sum of all the other phyla/genera, excluding the top 10 phyla/genera.

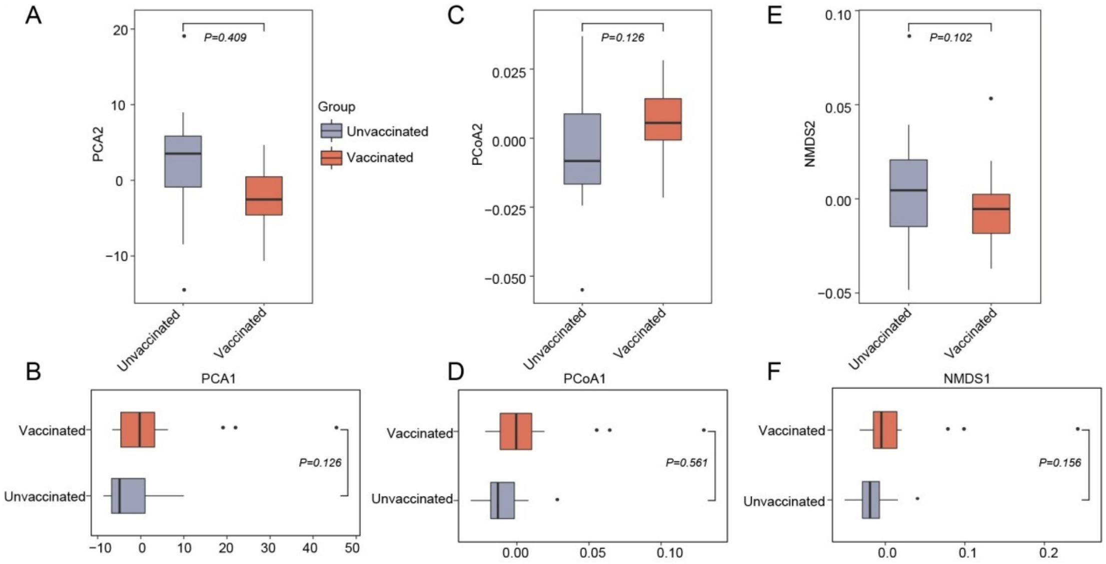

Figure S4. Assessment of the distribution of the β-diversity plots at axes based on the relative abundance of KEGG pathways in unvaccinated and vaccinated individuals. (A-B) Scatter plots distribution of β-diversity at the first and second PCA axes. P=0.126 for PCA1 axis; P=0.409 for PCA2 axis. (C-D) Elevation of the sample distribution at the first and second PCoA axes. P=0.561 for PCoA1 axis; P=0.126 for PCoA2 axis. (E-F) Comparison of the separation of each group at the first and second NMDS axes. P=0.156 for the NMDS1 axis; P=0.102 for the NMDS2 axis. The boxes represent the interquartile ranges, the inside lines represent the median, and the points are outliers. P-values were obtained using pairwise Wilcoxon tests.

Table S1. Data production of the study cohort.

Group	Sample ID	Raw PE(#)	Raw Tags(#)	Clean Tags(#)	Effective Tags(#)	Base(nt)	Avglen (nt)	Q20	Q30	GC%	Effective %
Unvaccinated	Unvaccinated-1	151,881	148,481	126,404	121,645	53,482,439	439	98.81%	95.59%	53.22%	80.09%
	Unvaccinated-2	158,961	154,448	146,629	138,561	57,710,807	416	98.40%	94.58%	53.14%	87.17%
	Unvaccinated-3	108,198	105,444	100,518	97,573	41,033,861	420	98.55%	94.88%	53.44%	90.18%
	Unvaccinated-4	142,179	138,297	132,119	127,604	53,681,792	420	98.43%	94.52%	51.12%	89.75%
	Unvaccinated-5	155,290	151,164	143,791	135,522	56,597,934	417	98.43%	94.61%	54.14%	87.27%
	Unvaccinated-6	79,471	77,239	73,621	70,163	29,072,041	414	98.41%	94.51%	53.60%	88.29%
	Unvaccinated-7	184,965	177,578	165,061	156,959	63,444,127	404	98.16%	93.65%	54.11%	84.86%
	Unvaccinated-8	174,875	170,437	161,825	158,598	66,616,916	420	98.53%	94.75%	53.48%	90.69%
	Unvaccinated-9	80,338	78,486	71,223	70,484	30,315,202	430	98.64%	95.10%	52.84%	87.73%
	Unvaccinated-10	171,673	166,814	158,949	152,744	63,605,358	416	98.40%	94.50%	53.30%	88.97%
	Unvaccinated-11	138,472	135,012	128,696	123,844	51,896,548	419	98.48%	94.65%	52.85%	89.44%
	Unvaccinated-12	101,496	99,531	94,966	89,318	37,655,838	421	98.58%	94.93%	52.79%	88.00%
	Unvaccinated-13	130,400	127,602	109,029	105,428	46,304,033	439	98.82%	95.62%	52.77%	80.85%
	Unvaccinated-14	164,469	159,532	133,193	125,884	55,289,058	439	98.65%	95.19%	52.92%	76.54%
	Unvaccinated-15	145,755	142,081	134,883	132,470	55,564,027	419	98.47%	94.72%	53.85%	90.89%
	Unvaccinated-16	146,214	141,565	133,165	128,517	53,418,632	415	98.32%	94.24%	54.25%	87.90%
	Unvaccinated-17	131,088	127,101	120,027	117,164	48,567,037	414	98.33%	94.33%	53.70%	89.38%
	Unvaccinated-18	176,407	172,685	164,948	152,996	64,519,522	421	98.57%	94.87%	52.54%	86.73%
	Unvaccinated-19	166,076	162,551	154,867	148,144	62,628,776	422	98.56%	94.85%	51.94%	89.20%
	Unvaccinated-20	116,013	113,693	108,410	101,728	42,968,280	422	98.57%	94.80%	52.20%	87.69%
Vaccinated	Vaccinated-1	74,259	73,603	69,779	62,238	26,913,427	432	98.33%	97.46%	52.61%	83.81%
	Vaccinated-2	53,294	52,858	47,821	46,341	20,263,686	437	97.62%	96.23%	52.95%	86.95%
	Vaccinated-3	63,757	62,114	57,798	56,651	23,390,006	412	96.86%	93.33%	53.09%	88.85%
	Vaccinated-4	60,550	59,878	55,358	54,841	24,235,892	441	98.03%	95.63%	52.40%	90.57%
	Vaccinated-5	47,019	46,358	42,243	40,003	16,843,021	421	96.72%	94.89%	52.87%	85.08%
	Vaccinated-6	62,291	61,372	58,047	52,675	22,445,514	426	97.44%	96.15%	52.19%	84.56%
	Vaccinated-7	73,183	72,235	68,149	64,109	27,713,513	432	97.95%	96.92%	52.05%	87.60%
	Vaccinated-8	91,038	90,423	86,073	75,439	33,169,042	439	98.67%	97.88%	53.36%	82.87%
	Vaccinated-9	81,527	80,946	77,076	69,547	30,353,239	436	98.75%	98.02%	54.36%	85.31%
	Vaccinated-10	78,192	77,483	73,267	66,502	29,499,739	443	98.42%	97.51%	54.67%	85.05%
	Vaccinated-11	87,882	87,194	82,499	77,366	34,373,545	444	98.64%	97.80%	55.44%	88.03%
	Vaccinated-12	89,023	88,365	84,437	71,629	31,018,019	433	98.46%	97.63%	53.28%	80.46%
	Vaccinated-13	117,645	116,064	111,699	107,722	45,959,445	426	98.68%	95.23%	53.49%	91.57%
	Vaccinated-14	67,535	66,791	63,428	58,414	25,462,120	435	98%	96.88%	54.13%	86.49%
	Vaccinated-15	100,787	99,919	95,144	83,970	37,072,973	441	98.40%	97.47%	54.67%	83.31%
	Vaccinated-16	92,242	90,882	86,470	83,630	35,526,005	424	97.65%	96.41%	53.42%	90.66%
	Vaccinated-17	100,557	99,682	94,528	87,322	37,865,627	433	98.48%	97.66%	53.23%	86.84%
	Vaccinated-18	112,463	110,934	105,487	97,534	41,679,947	427	97.73%	96.56%	52.58%	86.73%
	Vaccinated-19	47,773	46,935	42,815	40,560	16,801,489	414	96.11%	94.02%	52.32%	84.90%
	Vaccinated-20	48,747	48,191	43,576	42,420	18,337,617	432	97.46%	96%	52.92%	87.02%
